# An awareness-raising framework for global health networks: lessons learned from a qualitative case study in respectful maternity care

**DOI:** 10.1186/s12978-018-0662-9

**Published:** 2019-01-08

**Authors:** Meaghan O’Connor, Kayla McGowan, R. Rima Jolivet

**Affiliations:** Maternal Health Task Force, Women and Health Initiative, Harvard T.H. Chan School of Public Health, 651 Huntington Avenue, Boston, MA 02115 USA

**Keywords:** Respectful maternity care, Disrespect and abuse, Global health networks, Awareness raising, Case study

## Abstract

**Background:**

The increase in global health networks as mechanisms for improving health and affecting social change has been recognized as a key component of the global health landscape today. To successfully raise awareness of shared causes, global health networks need tools to help them plan successful campaigns and evaluate the impact of their work, as well as to coordinate and reinforce each other’s efforts. One global health network, the Respectful Maternity Care (RMC) Global Council, can be credited with raising the profile of the issues of disrespect and abuse (D&A) in childbirth and the need for RMC within global maternal health. We set out to learn from the work of the RMC Global Council and the RMC movement at large to develop a tool—a framework for planning and evaluating awareness-raising efforts—useful for networks focused on global health and human rights.

**Methods:**

We reviewed the literature for theoretical models on awareness raising and, finding a lack of appropriate tools, developed a new, draft framework using components of the Framework for Effective Campaigns, the SpitFire SmartChart 3.0, and Network Theory. We conducted semi-structured interviews with members of the RMC Global Council to validate the draft framework and identify any additional strategies or tactics that were used during their efforts to raise awareness of D&A and RMC. We also interviewed “influenced” individuals to validate inputs from the influencers and determine the key documents, events, individuals, and organizations that made the greatest contribution to the increased awareness of D&A/RMC. Data were analyzed using deductive and inductive qualitative research methods.

**Results:**

The validated awareness-raising framework includes five strategies that characterize a successful awareness-raising effort. Each strategy has a set of tactics that can operationalize those strategies. Each tactic is classified as essential, helpful, or variable based on the number of key informants who utilized it.

**Conclusion:**

This case study offers an example of how global health networks can create a movement that effects change at global and local levels by providing an empirically-grounded framework to help plan, coordinate, and evaluate future campaigns designed to raise awareness and create momentum in global health, human rights, and quality of care.

**Electronic supplementary material:**

The online version of this article (10.1186/s12978-018-0662-9) contains supplementary material, which is available to authorized users.

## Plain English summary

Today, networks of organizations and individuals are working together to meet global goals and improve health worldwide. These networks need tools to plan, coordinate, and evaluate their efforts in the most effective way. The global Respectful Maternity Care (RMC) movement, launched by the RMC Global Council, is an example of a network that worked together and effectively raised awareness of the mistreatment of women in childbirth facilities and women’s right to high quality, respectful care before, during, and after childbirth. To learn from this success, we used a case study approach to develop and validate a framework for awareness raising. We reviewed the literature on awareness raising and developed a new, draft framework drawing from the Framework for Effective Campaigns, the SpitFire SmartChart 3.0 tool, and Network Theory. We interviewed members of the RMC Global Council to compare their efforts to the items in our draft awareness-raising framework and note any additional strategies or tactics that they used. We also interviewed individuals who were influenced by the RMC movement to identify the key documents, events, individuals, and organizations that contributed to their increase in awareness. Data were analyzed using qualitative methods. The result of the research is an awareness-raising framework with five strategies for a successful awareness-raising effort. Each strategy has a set of tactics, labeled integral, helpful, or variable for awareness-raising efforts. Global health networks in many fields can use this tool when planning and assessing awareness-raising efforts.

## Introduction

Over the last seven years, the issues of disrespect and abuse in childbirth (D&A) and the need for respectful maternity care (RMC) have become key components of the global maternal health agenda. Much of the work that brought these issues to light was guided by the RMC Global Council, a network of individuals and organizations working collaboratively to raise awareness of D&A and RMC through research, programming, and advocacy. Today, global health networks like the RMC Global Council are more common than ever, bound together by “a common purpose oriented towards social change” and “durable relationships among the actors who constitute them” [1]. Indeed, Shiffman et al. write that “the proliferation of global health networks represents one of the most dramatic shifts in global health governance over the past three decades” [1]. To successfully raise awareness of shared causes—in particular those related to norms, such as respectful care—however, networks need tools to help them plan and coordinate their work, ultimately allowing them to reinforce rather than duplicate each other’s efforts [2]. Furthermore, monitoring and evaluating awareness raising has historically been a challenge for researchers and program implementers, and work is needed to provide tools to track progress and impact in this area.

Health messages that reach the entire spectrum of stakeholders at once—from patients, caregivers, and health facility managers, to local, state, national, and global policymakers—versus those that just reach a single group of stakeholders are more likely to raise awareness of an issue [3], but few organizations have the resources to implement such a comprehensive undertaking. A network of organizations and/or individuals can pool its resources and tap into its individual connections to facilitate efforts that can reach beyond the capacity of a single actor [4, 5]. However, unlike individual organizations or formal alliances guided by a specific contract, network members nearly always participate on a voluntary basis and leadership is often spread out among the members [1]. Thus, guiding frameworks and tools are especially important when many groups are working together, bringing different resources and perspectives to the table.

The RMC movement, catalyzed by the RMC Global Council, provides a particularly interesting case for examining and learning from the work of a global health network. The council, formed in 2011, now includes more than 300 individuals from around the globe, all of whom have a keen interest in advancing and protecting the rights of childbearing women. The council meets regularly and engages in advocacy and information-sharing globally and locally. We set out to learn from the success of the RMC Global Council and the RMC movement it helped engender to develop a tool—a framework for awareness raising—useful for networks focused on global health and human rights.

### Raising awareness

Networks can play a key role in raising awareness of an issue, which is often the first step in catalyzing change. Awareness messages “provide general background … and can be used to reinforce the importance of informed action and behavioral change” [6]. Many issues in global health—including maternal health—have gained traction through the efforts of networks [3, 4, 7]. A historic document, a landmark event, an engaging or powerful champion, and the persistence of many dedicated advocates can direct attention to an issue, raising both awareness and resources. A recent systematic review of efforts to raise awareness of maternal health rights found that “efforts that pursued multi-level, stakeholder and sectoral pathways were likely to build synergies that sustain the promotion of rights over the long term” [5].

Nevertheless, evaluating the effectiveness of awareness-raising activities is challenging, particularly in the case of policy and advocacy and when traditional media (e.g. television, radio) with existing standards for measurement are not employed. A 2014 high-level convening of the Global Policy & Advocacy and Measurement & Evaluation teams at the Bill & Melinda Gates Foundation (BMGF) emphasized that when evaluating awareness related to policy and advocacy, “the field … is still evolving” and “the focus should be on contribution, not attribution” [8]. Interestingly, Shiffman identifies a similar challenge when describing the difficulty of attributing change to the work of a network: “Detecting network influence is more difficult as one moves from outputs to policy consequences to impact” [1]. The consensus that emerged from the high-level convening was that monitoring and evaluation (M&E) practices, such as the application of behavior change theories and the development of indicators, can provide structure to awareness-raising efforts that can aid in tracking progress [2, 8].

Focusing on contribution rather than attribution, we can use at least two “tracer” indicators to track the success of the RMC Global Council and its members’ work to raise awareness of RMC: research papers and news articles published and national dialogues conducted. These tracers can signal the contributions of the council and its members’ work. A PubMed search for “respectful maternity care,” “disrespect and abuse,” or “obstetric violence” published between 2000 and 2009 results in zero papers, while the same search terms yield 82 papers on the topic published in 2010 or later. Indeed, more than half of those papers were published within the last two years. In popular culture, a Google News search for “respectful maternity care” articles published between 2000 and 2009 again finds zero articles, while the same search results in 42 news articles published since 2010. The specific search strategy can be found in Table 1.

**Table 1:** “Respectful maternity care” 1 publications search strategy

PubMed	
*(“respectful maternity care”[All Fields] OR “disrespect and abuse”[All Fields]) OR “obstetric violence”[All Fields] AND (“2000/01/01”[PDAT] : “2009/12/31”[PDAT])*	
*(“respectful maternity care”[All Fields] OR “disrespect and abuse”[All Fields]) OR “obstetric violence”[All Fields] AND (“2010/01/01”[PDAT] : “2018/3/29”[PDAT])*	
Google News	
*“respectful maternity care”[All Fields] AND (“2000/01/01”:“2009/12/31”)*	
*“respectful maternity care”[All Fields] AND (“2010/01/01”:“2018/3/29”)*	

Furthermore, since 2010, RMC has been a topic of national dialogue among law- and decision-makers in Nigeria, Afghanistan, Nepal, and Malawi, resulting in the specific inclusion of RMC in laws or professional standards [9–13]. Taking this as evidence of an increased awareness of D&A and RMC within the period in which the RMC Global Council was active, we looked for explanatory frameworks that could help explain *how* RMC awareness-raising efforts led to the observed impact. Our search for tools and frameworks for raising awareness yielded topic-specific tools and toolkits with indicators and social or behavioral change theories [14–17] that are not necessarily applicable to other health issues or audiences. For example, toolkits for raising awareness on managing household mercury waste [18], literacy in Europe [19], and gender roles and political participation in the Middle East and North Africa [20] do not share common measures of success or even social or behavioral change theories. Thus, a gap remains, leaving a need for a broader tool that can help networks to effectively raise awareness of any topic across many stakeholder groups.

### Disrespect & Abuse/Respectful Maternity Care

D&A—also known as “mistreatment” or “obstetric violence”—includes physical, verbal, and sexual abuse, violations of privacy, confidentiality, and/or consent, and detainment of the woman or her baby for failure to pay for the services provided at a facility [21–23]. Childbirth is a uniquely vulnerable time, and what happens during this period can have lasting effects on the health and wellbeing of both mother and baby. RMC applies human rights principles to maternity care to ensure that all women and their newborns receive high-quality interpersonal care throughout the childbearing process. As the White Ribbon Alliance (WRA) describes, RMC is a “universal human right due to every childbearing woman in every health system” [24].

In the 1970s, women’s rights advocates first exposed cases of women being disrespected or abused in health facilities during labor and delivery [22]. In the 1990s, work by Rachel Jewkes [25] in South Africa and the primarily-Latin American movement to combat *violencia obstetrica* (obstetric violence) [26–28] continued to underscore the need for increased attention to women’s rights during childbirth. Later, as more women around the world were encouraged to seek facility-based care during pregnancy and childbirth [29], research thrust provider behaviors and facility and health system failures into the spotlight [30]. Despite this important work, the global magnitude and significance of D&A remained a silent, veiled issue. Anecdotally, many providers and pregnant or postpartum women expressed familiarity with such injustice, but the lack of public awareness about D&A and its effect on utilization of facilities led to continued silence and mistreatment [30–32].

In 2010, the United States Agency for International Development (USAID) allocated funding towards research and advocacy on the subject, convening members of the global community to initiate a concerted effort against D&A. USAID initially commissioned the seminal paper, “Exploring Evidence for Disrespect and Abuse in Facility-based Childbirth: Report of a Landscape Analysis,” by Diana Bowser and Kathleen Hill [30], which was followed by funding for projects in Kenya and Tanzania to study the prevalence and drivers of D&A and design tailored interventions to address it, led by the Population Council and the Averting Maternal Death and Disability (AMDD) program at Columbia University, respectively [33–35]. USAID-funded advocacy activities, led by the WRA, centered on convening stakeholders, developing strategies, and undertaking activities that would raise awareness of D&A and the need to provide RMC for all women. To do so, WRA formed the RMC Global Council, whose first joint action was the development of “Respectful Maternity Care: Universal Rights of Childbearing Women,” more commonly referred to as the “RMC Charter,” which was released in 2011 [36]. Since then, the council has encouraged research into and dialogue around RMC by conducting a global survey on RMC, creating a publicly-available wiki page online to act as a repository for RMC resources, and releasing briefs on RMC in different contexts, among other activities [37].

In concert with the formation of the RMC Global Council and the momentum created by the studies in Kenya and Tanzania, several other programs and activists began to adopt RMC-related work. USAID continued to fund research as well as implementation programs through the Maternal and Child Health Integrated Program (MCHIP) and, later, the Maternal Child Survival Program (MCSP). But what was it about these activities—and subsequent events—and the individuals and organizations driving the movement that helped land RMC on the global and national maternal health agenda?

The RMC Global Council’s work from 2010 to 2016 provides an illustrative example of how a global health network can successfully raise awareness of an issue across multiple groups of stakeholders, from individual women seeking maternal health care to the global organizations that set the agenda for millions. The purpose of this case study is to learn from the experiences and successes of the RMC Global Council to inform the development of a validated, empirically-grounded strategic framework that outlines the critical success factors for effectively raising awareness of a policy- and human rights-related issue. Although the framework is likely most effective when employed by a network, many of its components also apply to individual activists.

## Methods

### Case study approach

To identify the critical success factors that raised the visibility of and concern for RMC within the global maternal health agenda, we selected a qualitative case study approach. Case studies are well suited to examine a real-life phenomenon in depth and in context [38]. Given that the work of the RMC Global Council was executed by numerous organizations and individuals and was intended to reach a broad audience, the case study approach is an appropriate method for capturing and compiling the various strategies and tactics used by the different council members and organizations into a coherent framework. Furthermore, to our knowledge there are no other close examinations of awareness raising in the context of maternal health and human rights. The case of RMC is thus unique, as is our approach of examining the phenomenon of RMC from the perspective of both those implementing the awareness-raising efforts—the “influencers”—and those affected by the efforts—the “influenced.”

### Framework development

We reviewed the literature on theories and frameworks for raising awareness in public health, human rights, and communication to guide the development of study tools. Given the absence of one theoretical model or tool that provided a suitable explanatory framework for the case of D&A/RMC movement, we combined three relevant resources into a new draft framework for raising awareness: the Framework for Effective Campaigns (FfEC) [39], the Spitfire SmartChart 3.0 tool (Smart Chart) [40], and Network Theory [41]. The FfEC is not a formal theory but provides recommendations for developing public information campaigns meant to influence decision-makers at the policy level. Given that addressing D&A and normalizing RMC requires changes at the interpersonal as well as policy level, this framework’s recommendations—encompassing multiple perspectives—are particularly relevant to the case at hand. The SmartChart is a tool to help nonprofit organizations create effective, strategic communication campaigns. WRA used the SmartChart to guide its advocacy strategy development during the period under study, thus making it a pertinent tool against which to evaluate the case of RMC. (Note that for the purposes of this paper, we defined “campaign” as organized efforts intended to achieve a common goal with a focus on awareness raising, including but not limited to advocacy, public information, or communications.) Finally, Network Theory examines the way associated individuals and groups interact, partner, and share resources with one another in order to achieve individual and shared goals. This mix of resources was chosen strategically: because of the multi-disciplinary, multi-stakeholder nature of the RMC Global Council—and the varied approaches the council members took to awareness raising (e.g. research, communications, and policy advocacy)—it made sense to cast a wide net and consider scholarly frameworks, job aids and tools, and broader social theories, in order to capture the complexities of such a diverse network. Together, these three resources address the spectrum of characteristics underpinning the RMC awareness-raising effort: policy change, formal communication planning, and strategic partnerships and networks. Using the FfEC, the SmartChart, and Network Theory, we drafted a theoretical framework—using language from the three resources—for raising awareness across multiple audience levels to be validated with data gathered through qualitative research methods. Some of the elements can be found in more than one of the resources (e.g. “define and describe the target audience”), while others are unique to a particular source or are a modification of an existing element. Table 2 outlines the different elements and indicates the resource from which it was derived.

**Table 2:** Draft awareness-raising framework

*A successful awareness-raising effort …*	FfEC	SmartChart	Network Theory	New
...is supported by a clearly-defined strategic plan.				
Define your position		X		
Define the ultimate goal		X		
Assess (continually) the current context and environment				X
...targets the right audience and aims to capture its attention.				
Define and describe the target audience, including their readiness and core concerns	X	X	X	X
Select appropriate communication channels and expose the audience sufficiently	X	X		
Stand out and be noticeable	X			
...delivers a credible, understandable message.				
Select a credible source and messenger	X	X	X	
Craft a clear message	X			
Fit the message with prior knowledge and the current context and environment	X			X
...delivers a message that influences the audience.				
Provide information	X		X	
Direct attention	X			
Trigger norms	X		X	
Change underlying values and/or preferences	X			
Select overall theme and tone of message		X		
Expose the audience sufficiently		X		
...shapes contexts and environments.				
Leverage the problems and pressures that govern behavior	X			
Involve the audience	X			
Use multiple channels	X			
Coordinate with like-minded partners/organizations/individuals			X	X
...is supported by sufficient resources.				
Identify and allocate human and financial resources		X		

FfEC: Framework for Effective Campaigns

SmartChart: Spitfire SmartChart 3.0

Network Theory: Network Theory

### Study participants

Study participants were categorized into the following two groups: the “influencers”—or those who implemented the awareness-raising efforts—and the “influenced”—those who were affected by the efforts. An initial list of influencers was generated through purposive sampling using the list of names on the RMC Charter [42]. The influencers included researchers, program implementers, human rights specialists, legal advocates, educators, and individuals associated with donor agencies. The list of influenced individuals included those who have initiated and/or participated in RMC-related efforts at global, national, and local levels, such as researchers, program implementers, government officials, and community activists. This list was generated using snowball and purposive sampling. Theorizing that the council’s global work would drive national changes, we asked several members of the RMC Global Council to recommend individuals who have pushed RMC at a national level with whom we could speak (snowball sampling). We also used a Google search to identify professional organizations and researchers engaged in D&A/RMC-related work (purposive sampling). Interviews with the influenced individuals assessed the effectiveness of the activities and efforts undertaken by the influencers. Although many of the influenced individuals later became influencers themselves, the two groups were divided based on whether they participated in the development of the RMC Charter, given the charter’s role as one of the first group efforts of the RMC Global Council. Ethics approval for this study was granted by the Harvard T.H. Chan School of Public Health Office of Human Research Administration.

### Data collection

Data were collected using semi-structured key informant interviews that were conducted via online conferencing platform and directed by interview guides (see Additional files 1 and 2). During the interviews, influencers and the influenced individuals provided details of their individual activities and interest in D&A/RMC as well as the activities or approaches of the organizations with whom they were working during the study period. The interview guide for influencers organized questions around each of the components of the framework for raising awareness with the aim of validating those components (by identifying whether the component was utilized, or “present”), extracting any additional themes, and identifying the events, individuals, organizations, and documents that both participant groups considered most impactful over the study period. The questions included in the interview guide for influenced individuals aimed to identify:

- the messages being sent by the influencers and their respective organizations;
- the appealing aspects of the D&A/RMC-related messages; and
- the messengers, events, and documents that were particularly influential in raising their awareness of D&A/RMC.

For consistency, one member of the study team (MO’C) conducted the interviews and took detailed notes; interviews were recorded for reference only. Prior to commencing the interviews, each study participant provided informed consent which was captured in the notes and on interview recordings. Two members of the study team (MO’C and KM) analyzed the interview notes using deductive and inductive thematic coding. First, the interview notes were coded to identify those components of the draft awareness-raising framework that were present, not present, or not mentioned in each transcript. Next, inductive thematic analysis was used to identify any themes not included in the framework. The emergent themes were discussed and refined. All data and themes were analyzed using Nvivo 11.4.1 software (QSR International, USA). Prior to commencing analysis as a whole, the study team selected three sets of interview notes, coded them each independently, and compared the results to test inter-rater reliability. Once the study team reached an inter-rater reliability greater than 80%, the remaining 25 sets of interview notes were compared to the framework and coded. The study team also noted the events, documents, and individuals that each informant identified as being most influential in raising awareness of D&A and advancing the RMC agenda.

## Results

Twenty-eight individuals were interviewed, including 20 “influencers” and eight “influenced.” The influencers came from research and academia, health professional organizations, non-governmental organizations, government and donor agencies, human rights law, program implementation, and advocacy. The influenced individuals represented six countries and worked in the fields of research and academia, health professional organizations, non-governmental organizations, and government organizations. Data from the interviews informed the refinement and validation of the framework for awareness raising.

### Primary results: Validated framework for awareness raising

The final, validated framework for raising awareness includes five strategies that characterize a successful awareness-raising effort.

1. The effort utilizes elements of strategic planning
2. The effort aims to capture the attention of key stakeholders
3. The effort delivers a consistent persuasive message
4. The effort creates a receptive environment
5. The effort maximizes all existing and potential resources

Within each strategy is a set of tactics—framed as actions—that can operationalize the strategy. The resulting 20 tactics are organized into three groups based on the proportion of key informants who described using a component in their work: integral (reported by 80% or more influencers), helpful (reported by 50–79% of influencers), and variable (reported by fewer than 50% of influencers). See Table 3 below.

**Table 3:** Validated awareness-raising framework

*A successful awareness-raising effort …*	Score	Integral	Helpful	Variable
...utilizes elements of strategic planning.				
Clearly define your position on the topic	.90	X		
Clearly define your end goal or objectives	.75		X	
Constantly assess and adapt to the environment and context in which you are working	.95	X		
...aims to capture the attention of key stakeholders.				
Clearly define the target audience	.95	X		
Identify and pay attention to the different levels of influence (take a multi-level approach to identifying and approaching the target audience)*	.65		X	
Use communication channels that are appropriate given your target audience’s preferences and behaviors	.90	X		
Find ways to stand out and be noticeable	.25			X
...delivers a consistent, persuasive message.				
Ensure that your message is delivered by a credible messenger and uses credible sources	.95	X		
Ensure that your message is clear	.90	X		
Ensure that your message aligns with the target audience’s prior knowledge and the current context	.85	X		
Establish a shared, consistent language/lexicon, particularly when working with a loose coalition*	.70		X	
Ensure that the message is informative	.75		X	
Use your message and tactics to lead the target audience’s attention to your topic	.50		X	
Leverage the norms that guide your target audience’s behaviors	.80	X		
Leverage the target audience’s values and preferences and find ways to change or shift those	.80	X		
Select a consistent and appealing theme or tone for your message	.85	X		
Ensure that the audience is exposed to the message sufficiently for it to sink in	.40			X
...creates a receptive environment.				
Leverage the problems and pressures that govern the audience’s behavior(s)	.85	X		
Involve the audience in your efforts to develop or spread the message	.60		X	
Use multiple channels to reach the audience	.85	X		
...maximizes all existing and potential resources.				
Coordinate with like-minded partners (individuals and organizations)	1.0	X		
Identify your internal and external human and financial resources and allocate them wisely	1.0	X		
Leverage individuals’ passion for the topic especially when the work is largely unfunded*	.70		X	

*Emergent theme

Overall, 14 of the 20 elements in the draft framework were used by 80% or more of the influencers and can thus be considered integral to create a successful awareness-raising effort as they were constant across nearly all influencers regardless of setting, context, goal, or resource capacity. Of those 14 elements, eight were used by 90% or more of the influencers. For example, 90% (*n* = 18) of the influencers reported outlining a clearly defined position on RMC. Among those, the positions (of both individuals and organizations) fell into three categories: RMC as a human right, RMC as a quality of care issue, and RMC as a behavioral norm. A constant awareness of the current environment and willingness to adapt the effort and message accordingly was also integral (reported by 95% of influencers). Researchers and advocates took into account the existing legal, political, medical-cultural, and system factors/norms that might influence their audience’s beliefs and behaviors around pregnancy or childbirth. For example, one influencer working in Latin America explained that, “It was a favorable context [for RMC] in Bolivia, Ecuador, and Mexico. There was a strong consolidation of women’s groups and good media coverage of women’s/indigenous rights, and constitutional frameworks. It was a context in which there was accountability and awareness of the duty bearer’s responsibility.” Context shaped the way the influencers approached their work, and often, changes to goals or positions were in response to changes in the environment. Several respondents used the term “evolved” or “evolving” to describe the environment and context as well as their approach to promoting RMC.

Nearly all of the influencers (95%, *n* = 19) stated that they had a clearly defined target audience. Although not all influencers were concerned with the same audiences, the influencers reported targeting pregnant women, indigenous women’s groups, consumer groups, medical and midwifery students and practitioners, doulas, health care facility administrators, local parliamentarians, lawyers, academics, researchers, donors, and international development agencies. Some influencers focused on one audience while others found ways to reach multiple groups: one influencer explained, “[as an organization we] were trying to target maternity services worldwide. But as an obstetrician, I have been targeting medical education in personal work; with both medical students and qualified doctors as well as NHS midwives to make them aware of human rights in childbirth, respectful maternity care, and obstetric violence.” Influencers also reported using appropriate (i.e., relevant to the target audience’s information-consumption habits) communication channels (90%, *n* = 18) and employing credible messengers and sources (95%, *n* = 19) to share a clear message (90%, n = 18). “Using appropriate channels” meant attending and presenting at key maternal health technical meetings and human rights fora for some, or for others, creating posters on D&A/RMC and distributing them to health facilities. Credible messengers included leaders in maternal health from donor agencies (e.g. USAID), academia and research (e.g. AMDD), and professional organizations (e.g. IMBCO, DONA International).

For most of the influencers, the messages used were closely tied to each organization’s or individual’s position and centered on the basic concepts of human rights, respect for women (and providers), respect for culture and the variety of experience, and quality of care. The messages also differed slightly depending on the target audience in order to make the message as appealing as possible. As one influencer explained, “Because [our organization] works at so many levels (global, country-level and national and subnational levels) we strategically target our RMC messaging to specific audiences, using messages that we think will resonate with distinct stakeholders: [for example, we focus on] professional ethics for provider professional associations, human rights for women’s groups, increasing institutional delivery for policymakers, quality of care for Ministry of Health stakeholders, providers and clients, etc.” Another influencer working in research and implementation noted that her organization “had three levels of messages: policy level … health facility, and the community.”

Importantly, this research evaluated awareness-raising efforts that extended beyond traditional message dissemination. Because few of the influencers were engaged in formal communications campaigns, other actions such as funding programs or conducting research related to RMC conveyed the message that RMC was a priority. All influencers also reported identifying and strategically allocating human, financial, and material resources (100%, *n* = 20). Finally, all influencers reported partnering with other likeminded individuals and organizations (100%, n = 20), highlighting the importance of partnerships for successful awareness raising. As one influencer from an international development agency explained, “Coming from a health system perspective and structural perspective, when thinking about things that are non-health but that still affect health, you begin to put together a network [of organizations]. Nothing that’s important to be done can be done by a single group. You can’t change policy or be proactive alone, much less sustain those changes over time. We all need to be good colleagues and think about large ecological frameworks. Thinking about continuity is important, and partnerships are a part of that.”

Four of the 20 elements from the draft framework were used by 50–79% of respondents and can be considered “helpful” when creating and executing an awareness-raising effort. Defining an end goal or objectives (75%, *n* = 15), creating an informative (70%, *n* = 14) and attention-getting (50%, *n* = 10) message, and involving the target audience in the effort to raise awareness (60%, *n* = 12) were all helpful tactics for influencers. Participants described various ways in which they involved the target audience. One influencer videotaped providers during a birth and played it back for them so that they could see their own behavior—both respectful and disrespectful—firsthand. Another influencer described creating opportunities for dialogues between women and health providers “so they can exchange ideas about good practices … *best* practices” related to RMC.

Three additional elements—not included in the original draft framework—were identified through thematic analysis and fall into the “helpful” group: 1) identify and pay attention to the different levels of influence in maternity care (described further below); 2) use a shared, consistent lexicon around D&A/RMC; and 3) leverage individuals’ passion for RMC and human rights to support work when funding is low or nonexistent.

Sixty-five percent (*n* = 13) of the influencers noted that they identified and took into consideration the different levels of influence affecting maternity care when planning their work. These levels of influence include individual care providers, pregnant women, facility administrators, national decision makers, and global influencers, such as the World Health Organization (WHO). For example, one influencer explained that, “a big part for us is articulating a comprehensive strategy with different elements and targets—at the policy systems level, point of care with providers, and rights and demand perspective from women themselves.” On the other hand, an influencer whose work focused on research and facility-level solutions remarked that theirs was, “a very multipronged approach to look at whatever seemed to be a driver [of disrespect and abuse] in the facility.”

Seventy percent (*n* = 14) of respondents expressed the importance of having a shared language or lexicon regarding D&A/RMC among all of the different organizations and individuals acting as influencers; by using the agreed-upon term “respectful maternity care,” influencers felt they could support and complement, rather than distract from, the work being done by partners and other likeminded advocates. One influencer noted that, “as the debate [about D&A] has become more refined, there’s been more clarity. ‘Respectful maternity care’ is a good way of framing it. And that became a catch phrase, or a good hook for what’s fundamentally a moral and ethical orientation.” Another influencer highlighted the importance of using the term consistently: “We developed our measurement [of D&A] based on Bowser and Hill’s work, but since [the WHO] paper on different groupings of mistreatment, we’re using that. And … we won’t stop calling it [respectful maternity care] now, because the movement is there.”

A passionate commitment to RMC, high-quality care, and women’s rights were identified as central to the effort to raise awareness of D&A/RMC. More than half of the influencers (*n* = 14) described themselves or their colleagues as “volunteers,” all driven by the same commitment to RMC and women’s health and rights in general. One influencer described herself and her network of colleagues working on RMC as “a coalition of the willing.” Another influencer explained: “We’re also part of what is a growing movement where we interact with many people with no expectation of money changing hands or formal engagement. We’re just working toward a shared goal.”

Finally, two of the 20 elements from the draft framework were used by fewer than half of the respondents and thus can be considered “variable.” One quarter of influencers reported that they tried to find ways to stand out and be noticeable; for example, one influencer cited a promotional book tour that provided an opportunity to meet with women and speak about respectful maternity care. While only 40% (*n* = 8) of influencers reported that they made an effort to expose the audience sufficiently to their message, such efforts may have been captured in another component of the framework (see “use multiple channels”).

### Secondary results: Insights from influenced individuals

The influenced individuals reported hearing RMC-related messages that were well aligned with the messages being shared by the influencers: that RMC is a quality of care issue, a human right, and/or a behavioral norm. The messages were appealing to the influenced individuals primarily because of alignment with their existing work or interests. Participants reported that they could easily connect the need for RMC to their work at various levels of the health system, for example, in prevention of mother-to-child transmission (PMTCT) of HIV, the Sustainable Development Goals and the WHO Quality of Care Network, and gender-based violence. The influenced individuals also noted that the emergence of a single term (“respectful maternity care”) from the global community was meaningful, but that it was important that influencers also allowed for tailoring the messages and materials to local languages and contexts. For example, one influenced individual explained that in the local context in which he works, “the direct translation of ‘disrespect and abuse’ [into the local language] isn’t the same—it’s much stronger—and [health care workers] don’t think they’re engaging in such harsh behaviors. So we say ‘lack of respect.’”

Influenced individuals also noted the key role that the engagement and partnerships of influential, trusted players in global maternal health played in encouraging involvement in the RMC movement. One researcher described a technical meeting on RMC featuring a number of international and national development organizations: “Everyone was sharing their experiences and the complexities of trying to define RMC and conduct research. And from that … is what launched [our organization’s] work in the area.”

### Validating influential events, documents, and individuals

**Fig. 1:**
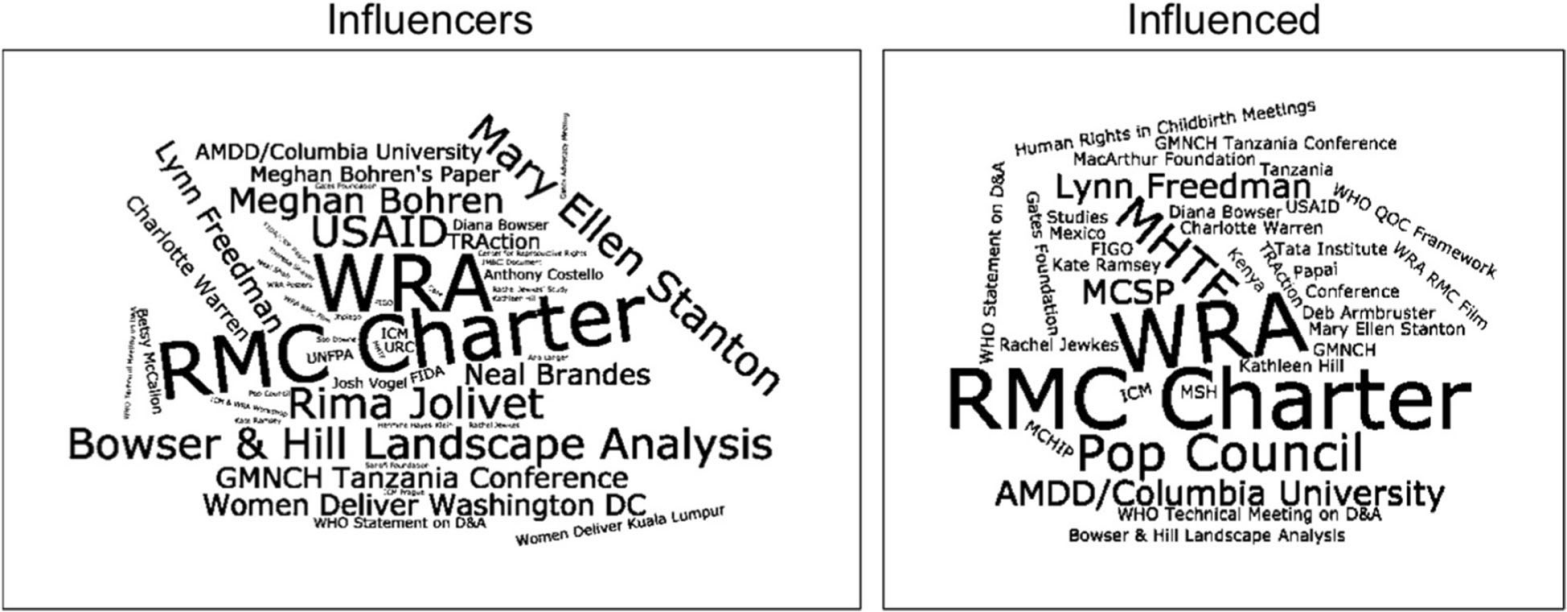
Word clouds depicting key individuals, organizations, documents, and events in the in the D&A/RMC awareness-raising effort, by key informant type

The research team also reviewed the interview transcripts to identify the individuals, organizations, documents, and events that both groups of respondents cited as instrumental in raising awareness of D&A and RMC. Using these data, we created two word clouds (see Fig. 1 below) that show the similarities and differences regarding what was considered most influential in raising awareness of D&A and RMC during the study period. For the influencers, the most influential documents, events, organizations, and individuals in the effort to raise awareness of D&A and the need for RMC tended to be at a higher global level. For example, many of the influencers cited the work of Bowser and Hill [30], as well as individual RMC champions at WHO and USAID. The events, documents, or individuals that the influenced participants felt were particularly influential in raising awareness of D&A/RMC, however, tended to be those that operated at a more local level, engaging the specific countries and communities in which they (the influenced participants) were working, such as the WRA, Population Council, and AMDD.

## Discussion

The results of this case study suggest that the increase in awareness of D&A/RMC can be attributed to the use of the five strategies and their accompanying tactics outlined in the validated framework described above. By utilizing these strategies and tactics, the RMC Global Council was able to plan wisely and use their resources effectively in order to share the right messages with the appropriate stakeholders and create an environment that was and is receptive to the concept of respectful maternity care.

Beyond identifying and validating specific, successful strategies and tactics for raising awareness, the results suggest that the respectful maternity care movement also benefited from the RMC Global Council taking an approach that valued consistency, flexibility, and collaboration.

Consistency in position, message, language, and target audience among the influencers helped to create a unified message despite the lack of a formal, overarching communications, public information, or advocacy campaign guiding the actors’ work. The influencers who chose to align D&A/RMC within human rights and quality of care positions linked to two larger current issues in maternal health, giving them the ability to utilize existing networks of allies, resources, and language. It also allowed them to embed their message into those of their allies, as evidenced by the data indicating that RMC was appealing to individuals and organizations working in various fields, from global maternal health directives to gender-based violence to PMTCT. Those whose position framed RMC as a behavioral norm were able to tap into the target audiences’ core values. Both positions also tied closely to what Smith and Shiffman describe as the existing global health community’s understanding of maternal survival as an issue of social justice and women’s rights [3]. The messages that influencers selected were closely tied to their chosen positions and centered on the concepts of human rights, respect for women (and care providers), respect for culture and the variety of experience, and quality of care. A relatively consistent use of the term “respectful maternity care” among multiple influencers likely helped create a stronger, more cohesive message. While RMC was not the only term used—others were selected based on local contexts—the use of this one, shared term reinforced the impression that this was a multi-partner, multi-stakeholder movement. Finally, a common understanding of the different target audiences—especially care providers and policymakers—who needed to hear the message meant that often, influencers’ messages converged or reached the same audience members at different points, reinforcing the message.

Flexibility within the influencers’ approaches to their D&A/RMC-related work also supported their awareness raising efforts. By maintaining a constant awareness of the context in which they were working and a willingness to adapt and evolve given that context, the influencers were able to maintain their relevance and level of persuasion regardless of environmental changes. Mechanisms and efforts to allow for tailoring via language (i.e., translating into local languages and commonly understood, contextually-relevant terms, such as “obstetric violence” or “mistreatment” versus “disrespect and abuse”)—while still complementing the shared lexicon—and message choice also supported that flexibility. The recognition that culturally appropriate language, materials, and channels must be employed highlights the importance of using the right words—in the right language—to make a message as clear as possible for the target audience. This is a similar finding to that of a recent review of principles and processes of promoting maternal health rights in which the authors describe this “process of vernacularism as being critical for local stakeholders to adopt rights principles” [5]. Several influencers also noted that they used different messages for different audiences. The purpose of this tactic was to ensure that each audience was receiving a message that would resonate most with them given their interests, concerns, prior knowledge, and values. Global health networks may benefit from the finding that while consistency—as noted above—is important for creating a sense of belonging to a larger movement, flexibility is equally as important to ensure that messages, materials, and approaches resonate with the community in which they are shared. The case of RMC is an insightful example of how networks can achieve balance, creating a movement to effect change at both the global and local levels.

Finally, collaboration among the influencers and their individual networks helped synchronize messages and allowed organizations to amplify their reach as well as complement and support shared goals. Organizational buy-in also created opportunities for more individuals to get involved, creating a mutually supportive environment and growing the community of RMC advocates. Every influencer used partnerships to gain access to resources and audiences they could not access alone. Partnerships allowed influencers to leverage the authority of trusted messengers and experts in other organizations as well as to lend individuals the authority of their and others’ backing organizations.

Jeremy Shiffman posits that global health networks face four primary challenges: problem-defining, positioning, coalition-building, and governance [7]. The RMC Global Council’s use of consistent messaging and a shared language with room for tailoring to specific contexts helped to overcome Shiffman’s first two challenges, and the council’s partnerships helped to overcome the challenge of coalition-building. Indeed, all of these factors may have also been supported by the strength of the existing maternal health network: both Shiffman and Smith (2016) note that the network of global health actors driving the maternal health agenda can be considered among the more successful examples of global health networks of the past few decades thanks to concerted efforts to clearly define the problem and agree on a position [3, 4, 7].

The findings of this case study should be considered in light of some methodological limitations. First, it relied solely on the recollections of individuals who acted as influencers and the influenced individuals, which are subject to memory or recall bias. Second, the sample size was limited, particularly in the case of the influenced individuals. Including more influenced individuals could have provided greater insight into how the messages shared by members of the RMC Global Council’s resonated in a wider variety of settings (e.g. other countries/regions, different types of organizations). Including more individuals overall—influencers and influenced—could have also provided an even more robust picture of the case, but this study was not designed to test saturation. Third, although the study team made an effort to consider awareness-raising resources from a variety of fields, the process to select the resources ultimately used to build the draft framework was inherently subjective and thus, other relevant theories, tools, or frameworks may have been overlooked. Finally, the data collection tools were structured around the awareness-raising framework, which used a number of communications concepts and terms (e.g. message, tone, communication channel) that seemed unfamiliar or irrelevant to several of the key informants given that their work was not part of a formal communications campaign or strategy. Indeed, one influencer noted that she and her colleagues “never saw [themselves] as raising awareness in the way that communications or advocacy people do” and reiterated throughout her interview that her organization’s work was not done with a communications mindset. Although analysis of the key informants’ responses showed that many of the respondents were indeed using the concepts included in the framework, the fact that the respondents did not recognize the terms used may have affected their understanding of and responses to the interview questions.

The validated framework describes the strategies and tactics needed for a successful awareness-raising “effort” that entails more than a traditional communications, public information, or advocacy campaign. As the case of D&A/RMC awareness raising demonstrates, not all individuals or organizations commenced their work with the express intention of raising awareness about D&A or RMC; several of the influencers represented research organizations exploring the causes of D&A in order to identify ways to promote RMC. Indeed, only one of the influencer organizations (WRA) we spoke with used a formal tool or theory (SmartChart) to guide their work. Often, raising awareness about D&A/RMC was embedded in research projects, programs, or training sessions on other maternal and newborn health, quality of care, and human rights topics. “Effort” is thus perhaps a more comprehensive term than campaign, and one that allows for the inclusion of a variety of activities and makes the resulting framework relevant for a broader audience. Furthermore, presented thus, this innovative framework outlining key strategies for successful awareness-raising efforts becomes a tool that networks can use to overcome some of the challenges of problem-defining, positioning, and coalition-building.

## Conclusion

This case study draws on the FfEC, SmartChart 3.0, and Network Theory to develop a new awareness-raising framework, and, in doing so, provides a fresh, new perspective on each of these existing resources. By linking these three resources together, we have created a robust new framework that allows networks of advocates to approach awareness raising from multiple angles and levels—including policy change, formal communication planning, and strategic partnerships and networks—simultaneously and address issues that others have identified as perennial challenges to networks’ success. As others have pointed out, awareness for awareness’s sake is not enough [43–45]. Because many of the members of the RMC Global Council were not focused specifically on awareness-raising as an end, but rather, a means to an end (such as informing research findings or policy change), raising awareness of D&A and the need for RMC was an important precursor to empowering women to speak up for themselves, changing laws and policies to promote RMC and protect women’s rights, and improving facility conditions and the quality of health care.

Thus, the results of this case study present two opportunities for networks. First, the RMC Global Council can learn from its own success as it strategizes future directions and next steps for ensuring every woman’s right to RMC (as well as providers’ rights to respectful working conditions). Second, and more broadly, other global health networks—and their respective researchers, program planners, and advocates—can utilize this validated framework, with its clear strategies and tactics, to help plan and evaluate future campaigns designed to raise awareness of neglected issues and in doing so, drive momentum in global health, human rights, and quality of care.

## Additional files

Additional file 1: Interview guide questions for influencers (DOCX 24 kb)

Additional file 2: Interview guide questions for influencers (DOCX 15 kb)

## Abbreviations

AMDD: Averting Maternal Death and Disability (Columbia University)
D&A: disrespect and abuse during childbirth
FFEC: Framework for Effective Campaigns
RMC: Respectful maternity care
USAID: United States Agency for International Development
WHO: World Health Organization
WRA: White Ribbon Alliance
