# Supplementary material for: An awareness-raising framework for global health networks: lessons learned from a qualitative case study in respectful maternity care
Source: Reprod Health. 2019 Jan 8;16:1. doi: 10.1186/s12978-018-0662-9 (PMC6323747; doi:10.1186/s12978-018-0662-9)
Supplement: Supplementary file 1 — Interview guide questions for influencers (DOCX 24 kb) [file 12978_2018_662_MOESM1_ESM.docx]

Supplementary File 1. Interview guide questions for influencers

Interview Guide: Influencer Questions and Corresponding Framework Categories

| Key Informant information | 1. I’d like to begin by asking you to please state your name, organization, and your role within your organization. 2. If this is not the same organization you were with when you began working on RMC, can you please tell me what that organization was and what your role was there? |
| --- | --- |
| Utilizes elements of strategic planning | Now, thinking about your role as <title> with <organization>, I’d like to learn more about the work that was done by <aforementioned organization> related to RMC awareness-raising between 2010 and 2016.   1. Did your organization outline a specific goal or set of objectives when you started working on RMC? And if so, what was it? 2. Did your organization have a specific position on RMC? If so, how would you describe it? By position, I mean a certain point of view or attitude towards RMC. 3. Has the goal changed at all over time?    1. (If yes) What were some of the factors that influenced that change? 4. Has the position changed at all over time?    1. (If yes) What were some of the factors that influenced that change? 5. Would you say that your organization’s work was guided by an overall strategic plan? |
| Captures the attention of key stakeholders | 1. Can you tell me about who you were trying to reach with your RMC work, and why you selected those people or organizations?    1. What about those people or organizations made them important to reach with your message?    2. Who or what helped you identify those people or organizations? 2. How exactly did you go about getting the attention of your target audience? What tactics or communication channels did you use? |
| Delivers a consistent, persuasive message | 1. What was your RMC message and how did you go about creating it?    1. Was the focus of the message “disrespect and abuse” or “respectful maternity care?” Or both?    2. Did your message include a call to action? If so, what was it? 2. Was there a specific person (or persons) that represented your organization’s position and delivered your RMC message, and if so, who was it?    1. How did you select that person? 3. How do you think the message you chose changed or did not change your target audience’s thinking on RMC?    1. How do you know your message changed or did not change your target audience’s thinking on RMC?    2. Did you appeal to a particular aspect of your audience’s concerns? If so, what aspect?    3. Do you think the location(s) where your message was delivered played a role? If so, which location(s)?   How would you describe the overall tone of your message? For example, some awareness-raising campaigns choose particular images or words that try to create a sense of urgency or evoke specific emotions. |
| Creates a receptive environment | 1. Many providers, policy makers, etc. are – or at least were – unfamiliar with the disrespect and abuse women face when seeking maternity care. As a result, asking those same stakeholders to adopt the attitudes and practices of RMC requires a bit of a shift in both thinking and behaviors. How would you describe the context in which you were working? 2. How did you go about working within the existing context/paradigms to re-shape the maternal health environment in a way that might be more receptive to the concept of RMC? What that something you thought about? |
| Maximizes all existing and potential resources | 1. Did you work with anyone else (individuals or organizations) to implement your RMC work?    1. (If yes) Were those partnerships formal or informal? By formal, we mean a partnership that is regulated by some sort of a memorandum of understanding or contract. An informal partnership is a collaboration that is not guided by an official document. 2. If you partnered with other individuals or organizations, how did their resources help or hurt your work? 3. How did your organization support its RMC work? Were there dedicated funds or individuals devoted to the work? |
| Additional insights and key activities/individuals | 1. Is there anything you or your organization wish you had done differently at that time to raise awareness about disrespect and abuse or RMC? 2. From your perspective, would you say that awareness about disrespect and abuse/RMC was successfully raised – why or why not?    1. (If yes) Can you point to any specific activities or individuals that stand out to you as having been particularly influential or important to that success? These activities or individuals do not have to be a part of your organization. 3. Is there anyone else you recommend I reach out to for an interview for this case study? |

Supplementary File 2. Interview guide questions for influenced

Interview Guide: Influenced Questions and Corresponding Framework Categories

| Key Informant information | 1. I’d like to begin by asking you to please state your name, your organization, and your role within the organization. 2. If this is not the same organization you were with when you began working on RMC, and which we have previously agreed to focus on, can you please tell me what that organization was and what your role was there? |
| --- | --- |
| *Individual Perspective* | |
| Captures the attention of key stakeholders  Delivers a consistent, persuasive message | Now, thinking about your role as <title> with <organization>, I’d like to learn more about your experience with RMC 2010 and 2016.   1. To the best of your knowledge, when did **you** first hear about either disrespect and abuse or RMC? 2. Was there something or someone specific that sparked your interest in disrespect and abuse/RMC? Perhaps an event, a speaker, or the release of a document?    1. What about that <event/speaker/document/experience/person> was so compelling to you? |
| Delivers a consistent, persuasive message  Creates a receptive environment | 1. What was the RMC or D&A message you heard?    1. Who did it come from? 2. What was it about the overall message you heard regarding RMC that appealed to you?    1. Was there anything else you were working on or involved in at the time that made RMC particularly appealing to you? What about current events? |
| Delivers a consistent, persuasive message | 1. Thinking back to that time, were there any individuals or organizations that were championing RMC, and if so who or what were they? 2. How did you interact with those individuals or organizations? By that I mean, did you work with them at all, and if so, how? |
| Additional insights and key activities/individuals | 1. So, once you were aware of disrespect and abuse/RMC, what were your next steps: What did you do with this information?    1. Have you personally done anything further to raise awareness of RMC? Or of disrespect and abuse in childbirth? |
| *Organizational Perspective* | |
| Captures the attention of key stakeholders | Now I want you to think about RMC from the perspective of your organization.   1. To the best of your knowledge, when did your organization first get involved in addressing disrespect and abuse or promoting RMC? 2. What has your organization done to address disrespect and abuse during childbirth or to promote respectful maternity care? (Laws, policies, additions to professional standards, etc.) 3. How would you describe your role in your organization’s decision to get involved in RMC? |
| Delivers a consistent, persuasive message | 1. What about RMC do you think appeals to your organization? 2. Are there any other individuals or organizations that contributed to your organization’s involvement in RMC? |
| Additional insights and key activities/individuals | 1. From your perspective, would you say that, overall, awareness about disrespect and abuse/RMC has been raised successfully – why or why not?    1. (If yes) Stepping outside your own perspective for a moment, can you point to any specific individuals or organizations that were especially responsible for that success? 2. Is there anyone else you recommend I reach out to for an interview for this case study? |
