# Supplementary material for: An awareness-raising framework for global health networks: lessons learned from a qualitative case study in respectful maternity care
Source: Reprod Health. 2019 Jan 8;16:1. doi: 10.1186/s12978-018-0662-9 (PMC6323747; doi:10.1186/s12978-018-0662-9)
Supplement: Supplementary file 2 — Interview guide questions for influencers (DOCX 15 kb) [file 12978_2018_662_MOESM2_ESM.docx]

Box 1. “Respectful maternity care” publications search strategy

**PubMed**

*("respectful maternity care"[All Fields] OR "disrespect and abuse"[All Fields]) OR "obstetric violence"[All Fields] AND ("2000/01/01"[PDAT] : "2009/12/31"[PDAT])*

*("respectful maternity care"[All Fields] OR "disrespect and abuse"[All Fields]) OR "obstetric violence"[All Fields] AND ("2010/01/01"[PDAT] : "2018/3/29"[PDAT])*

**Google News**

*“respectful maternity care”[All Fields] AND (“2000/01/01”:”2009/12/31”)*

*“respectful maternity care”[All Fields] AND (“2010/01/01”:”2018/3/29”)*
